# Gestational Age and Childhood Educational Outcomes in Primary and High School Years

**DOI:** 10.1001/jamanetworkopen.2026.31747

**Published:** 2026-09-01

**Authors:** Francisco J. Schneuer, Anthea Lindquist, Anna Forsythe, Parinaz Mehdipour, Jessica A. Atkinson, Antonia W. Shand, Adrienne Gordon, Andrew J. Martin, Raghu Lingam, Stephen Tong, Roxanne Hastie, Natasha Nassar

**Affiliations:** 1 Children’s Hospital at Westmead Clinical School, Faculty of Medicine and Health, University of Sydney, Sydney, New South Wales, Australia; 2 Leeder Centre for Health Policy, Economics and Data, Faculty of Medicine and Health, University of Sydney, Sydney, New South Wales, Australia; 3 Perinatal Epidemiology Group, Department of Obstetrics, Gynaecology and Newborn Health, University of Melbourne, Heidelberg, Victoria, Australia; 4 Mercy Perinatal, Mercy Hospital for Women, Heidelberg, Victoria, Australia; 5 Department of Maternal Fetal Medicine, Royal Hospital for Women, Randwick, New South Wales, Australia; 6 RPA Newborn Care, Royal Prince Alfred Hospital, Camperdown, New South Wales, Australia; 7 Charles Perkins Centre, University of Sydney, Sydney, New South Wales, Australia; 8 Reproduction and Perinatal Centre, Faculty of Medicine and Health, University of Sydney, New South Wales, Australia; 9 School of Education, University of New South Wales, Randwick, New South Wales, Australia; 10 Population Child Health Research Group, School of Clinical Medicine, University of New South Wales, Randwick, New South Wales, Australia

## Abstract

**Importance:**

Gestational age at birth plays a critical part in childhood neurodevelopment, especially for individuals born preterm (<37 weeks’ gestation).

**Objective:**

To investigate the association between gestational age and educational outcomes at primary and high school age, controlling for unmeasured confounding by family-level factors.

**Design, Setting, and Participants:**

This population-based cohort study conducted in Australia included all individuals born at 23 to 42 weeks’ gestation in New South Wales (NSW) between 2001 and 2011 and in Victoria between 2005 and 2012 who had school outcomes assessed by standardized assessments in grade 3 (age 8-9 years) and/or grade 9 (age 14-15 years). Analyses were conducted between June 2024 and February 2026.

**Exposure:**

Gestational age at birth.

**Main Outcomes and Measures:**

Standardized numeracy and reading assessment *z*-scores were calculated in grade 3 and grade 9 and compared across gestational age groups using multivariable general linear mixed models. A clinically meaningful difference was considered an adjusted mean difference (AMD) in *z*-score of 0.2 or greater.

**Results:**

The study included 1 095 816 children in grade 3 (772 589 NSW-born [50.7% male] and 323 227 Victoria-born [50.5% male]) and 292 027 NSW-born adolescents in grade 9 (50.7% male). Among children assessed at grade 3, 6.4% in NSW and 7.0% in Victoria were born before 37 weeks’ gestation and 54.1% in NSW and 41.1% in Victoria were born at 39 to 40 weeks’ gestation; 6.3% of NSW adolescents assessed at grade 9 were born before 37 weeks’ gestation and 53.8% at 39 to 40 weeks’ gestation. Compared with children born at 39 to 40 weeks, children born at 38 weeks had lower numeracy (AMD, −0.02; 95% CI, −0.03 to −0.02) and reading (AMD, −0.02; 95% CI, −0.02 to −0.01) *z*-scores in grade 3. *z*-Scores decreased in a stepwise manner for each gestational age group, with the greatest difference for those born at 23 to 27 weeks vs 39 to 40 weeks (numeracy: AMD, −0.54 [95% CI, −0.58 to −0.49]; reading: AMD, −0.29 [95% CI, −0.34 to −0.25]). However, among siblings at grade 3, these differences were attenuated, and clinically meaningful differences compared with birth at 39 to 40 weeks were only seen for numeracy in children born at 28 to 31 weeks (AMD, −0.23; 95% CI, −0.27 to −0.19) and 23 to 27 weeks (AMD, −0.45; 95% CI, −0.53 to −0.38) and for reading, only for children born at 23 to 27 weeks (AMD, −0.28; 95% CI, −0.36 to −0.21). Results were similar in grade 9, with clinically meaningful differences for numeracy (AMD, −0.53; 95% CI, −0.62 to −0.45) and reading (AMD, −0.39; 95% CI, −0.48 to −0.30) only seen for adolescents born at 23 to 27 vs 39 to 40 weeks’ gestation in the full-cohort analysis.

**Conclusions and Relevance:**

In this cohort study of over 1 million Australian children, the independent association of gestational age at birth with educational performance was most pronounced for children born at less than 28 weeks’ gestation; for children born from 28 to 38 weeks’ gestation, differences were not clinically meaningful and were likely primarily attributable to unmeasured confounding by shared familial factors. These findings support prioritizing prevention of the earliest preterm births and suggest that children born before 28 weeks’ gestation may benefit from early identification of learning difficulties and targeted educational support.

## Key Points

**Question:**

Is gestational age at birth associated with educational performance in childhood and adolescence after accounting for shared familial factors?

**Findings:**

In this cohort study of 1 095 816 children and 292 027 adolescents in Australia, lower gestational age was associated with poorer numeracy and reading scores. In sibling-comparison analyses, clinically meaningful differences were observed primarily among children aged 8 to 9 years born very preterm (<28 weeks’ gestation).

**Meaning:**

After accounting for shared familial factors, the findings suggest early preterm birth may be associated with poorer educational outcomes, whereas associations with birth at later gestational ages may be in part attributable to familial or environmental confounding.

## Teaser

This cohort study assesses the association of gestational age at birth with standardized reading and numeracy assessment outcomes in Australian children and adolescents at ages 8 to 9 years and 14 to 15 years.

## Introduction

Gestational age at birth is considered one of the most important predictors of childhood cognitive development. The fetal brain triples in size during the final trimester of pregnancy, with critical development continuing until birth.^1,2^ Brain development slows at birth, meaning that even after reaching term-equivalent age, individuals born prior to 40 weeks’ gestation show marked differences in brain structure and function.^3,4^ Those born at preterm (<37 weeks’ gestation) and early term (37-38 weeks’ gestation) have an increased risk of neurodevelopmental disability, cognitive delay, and poor academic performance when compared with their peers born at later-term gestations (39-40 weeks).^5,6,7,8,9,10,11,12,13^

Despite valid concerns about the impact of gestational age at birth, other factors can substantially influence long-term childhood neurodevelopment. The environment in which a child is raised plays a crucial role in determining educational, cognitive, and developmental outcomes. While previous studies investigating the association between gestational age at birth and educational and neurodevelopmental outcomes adjusted for a range of socioeconomic factors such as parental level of education and socioeconomic status,^5,6,7,8,9,10,11,12^ unmeasured family-level confounding may have remained. This confounding may include unmeasured socioeconomic, parental, genetic, and environmental factors that are difficult or impossible to measure directly.^14^ A 2023 article by Husby et al^15^ offered an elegant solution to this difficulty by performing a nested cohort study looking at the association of gestational age at birth with educational outcomes among siblings, thereby accounting for the potential confounding of unmeasured family factors. Comparing full siblings, the study showed that when the family environment was accounted for, educational outcomes in adolescence among children born between 34 and 39 gestational weeks did not differ from those born at 40 weeks.^15^

The findings of Husby et al^15^ may prompt a shift in historic thinking, suggesting that differences in educational achievement for individuals born before vs at or after 40 weeks’ gestation may also be attributed to differences in unmeasured family factors rather than solely gestational age. However, Husby et al^15^ assessed educational outcomes at 16 years of age, when effects may have dissipated, with preterm-born children having caught up with their peers by adolescence.^16^ It remains unknown whether differences in educational outcomes previously attributed to gestational age occur at ages earlier than adolescence and are also influenced by unmeasured familial confounding.^17,18^ Thus, we sought to investigate the association between gestational age and educational outcomes at primary school age (8-9 years) and high school age (14-15 years) among a multistate population-based cohort of Australian children, taking into account family-level confounding.

## Methods

This cohort study is reported as per the Strengthening the Reporting of Observational Studies in Epidemiology (STROBE) reporting guideline. Ethics approval for the study was granted in each respective state by the New South Wales (NSW) Population and Health Services research ethics committee and Mercy Health human research ethics committee. Each data custodian provided approval for data access and linkage. For the NSW cohort, data were linked by the NSW Centre for Health Record Linkage; for the Victorian cohort, the Centre for Victorian Data Linkage approved the project and performed the linkage. Given the retrospective and deidentified nature of this study, the requirement for individual participant consent was waived by the Australian Institute of Health and Welfare.

### Study Population

The study comprised 2 population-based cohorts of all children and/or adolescents born at 23 to 42 weeks’ gestation in the Australian states of NSW (individuals born from 2001-2011) and Victoria (individuals born from 2005-2012) who had corresponding school outcomes assessed by the National Assessment Program–Literacy and Numeracy (NAPLAN) in grade 3 (aged 8-9 years) and/or grade 9 (aged 14-15 years). NAPLAN assessments were conducted from 2008 to 2019 in the NSW cohort and 2013 to 2019 in the Victoria cohort. The grade 9 assessment was only available for NSW adolescents, with grade 9 data not available within the held Victoria dataset. Combined, NSW and Victoria represent approximately 57% of all individuals born in Australia.^19^

### Data Sources

Each participant’s information was obtained using each state’s administrative birth records linked to hospital admissions and Education Standards Authority NAPLAN records. Details of each data collection are included in the eAppendix in Supplement 1. Sibling pairs were identified using a common mother identifier number in the birth records.

### Study Exposure

The main exposure was gestational age at birth. This was measured in weeks and categorized as extremely preterm (23-27 weeks’ gestation), very preterm (28-31 weeks), moderately preterm (32-33 weeks), late preterm (34-36 weeks), early term (37-38 weeks), term (39-40 weeks; reference group), or late term or postterm (41-42 weeks’ gestation).

### Outcome Measures

Across Australia, standardized testing is performed in all mainstream schools in grades 3, 5, 7, and 9 via the NAPLAN, an educational assessment across 5 domains: grammar and punctuation, spelling, writing, reading, and numeracy.^20,21^ For this study, educational outcomes were based on NAPLAN numeracy and reading scores in grade 3 (age 8-9 years) and grade 9 (age 14-15 years—NSW only) among the study cohort. Each domain score was standardized as a *z*-score compared with the mean state result for the relevant numeracy or reading test and year of test. Students exempt from completing the NAPLAN test due to disability or chronic conditions do not receive a test score; thus, these individuals were only included in sensitivity analyses.

### Covariates

Maternal and birth characteristics previously reported to be associated with both gestational age at birth and school performance were considered potential confounders.^5,6,7,8,9,10,11,12^ These included maternal age at the time of conception (<25, 25-29, 30-34, or ≥35 years), any maternal smoking during pregnancy, diagnosis of preeclampsia, diagnosis of preexisting or gestational diabetes, parity, socioeconomic position (characterized by Socio-Economic Indexes for Areas quintile^22^), remoteness of residence (based on residential postcode and classified as major cities vs regional or remote areas), and parents’ highest achieved educational level (available only in NAPLAN data sources). Offspring characteristics included birth year and sex.

Additional data were ascertained for sensitivity analyses. These included a birth weight categorized as small for gestational age (SGA; <third percentile) or large for gestational age (LGA; >97th percentile), plural birth, major congenital anomalies recorded within hospital admission records from birth up to the first year of life (based on *International Statistical Classification of Diseases and Related Health Problems, Tenth Revision, Australian Modification [ICD-10-AM]* diagnosis codes, as per European Platform on Rare Disease Registration guidelines^23,24^), severe neonatal morbidity (based on birth data using a modified version of a composite neonatal adverse outcome indicator, created for use with administrative health data^25^), and days spent in hospital between birth and age 7 years.

### Statistical Analysis

An a priori statistical analysis plan was developed and agreed on by all authors. Analyses were conducted between June 2024 and February 2026. Maternal and neonatal characteristics were described by gestational age and by state (NSW or Victoria). Associations between gestational age and educational outcomes were first assessed by evaluating differences in mean *z*-scores for each gestational age group compared with the group born at 39 to 40 weeks’ gestation, using multivariable general linear mixed models for the full cohort. Next, to account for family factors, we restricted the cohort to siblings that were discordant for gestational-age group and reference group at birth and used a multivariable general linear mixed model with a fixed intercept, conditioning on the common maternal identifier. All multivariable models were adjusted for year of birth, sex, maternal age, smoking during pregnancy, parity, preeclampsia, preexisting or gestational diabetes, highest achieved parental educational level, socioeconomic disadvantage, and remoteness of residence, with models for grade 9 outcomes (NSW cohort only) also adjusted for grade 3 *z*-scores. Missing values for risk factors were coded and analyzed as a separate category if more than 2% of data were missing on a particular variable; otherwise, they were excluded from analyses.

Each respective model for numeracy and reading outcomes produced an adjusted mean difference (AMD), along with a 95% CI, in *z*-scores between gestational-age groups and the reference group (39 to 40 weeks’ gestational age). Although estimates with 95% CIs that excluded 0 were considered statistically significant, only results where the AMD was greater than plus or minus 0.2 SDs were considered clinically meaningful differences.^26^ When interpreting MDs, the Australian Curriculum, Assessment and Reporting Authority considers effect sizes (*z*-score MDs) of −0.2 to −0.5 to represent students with below-standard (reference) scores who require an action plan, while *z*-score MDs less than −0.5 are considered substantially below-standard (reference) scores, and students require an action plan, focused intervention, and additional support to help them achieve the skills they require to progress in schooling.^26^ Results based on multivariable general linear mixed models for each state were then pooled using inverse-variance–weighted fixed-effects meta-analyses, with pooled estimates for AMDs presented as summary effect sizes and 95% CIs. To assess the robustness of the findings, we also applied multivariable general linear mixed regression models to assess both between-children and within-family outcomes in the full cohort. This involved including an additional covariate of the family-level mean gestational age of all siblings to minimize confounding bias after accounting for the within-effect structure.^27^

We also conducted several sensitivity analyses for each test outcome and state. First, we included individuals exempt from NAPLAN by imputing their *z*-score equal to the lowest 1 percentile in numeracy and reading and repeating the analyses. Second, we stratified analyses by sex. Third, we conducted several restricted analyses by excluding individuals who were born SGA, individuals with major anomalies, individuals with severe neonatal morbidity, and individuals from a multiple birth. Fourth, we conducted an additional post hoc analysis assessing the association of gestational age with outcomes of adolescents who were below NAPLAN national minimum standards in grade 3 numeracy and reading tests. All analyses were conducted using SAS, version 9.4 (SAS Institute Inc), using GLM and GLIMMIX procedures, and the meta package for pooled analysis and plots in R, version 8.3-0 (R Project for Statistical Computing).

## Results

The study included 1 095 816 children (772 589 from NSW [49.3% female, 50.7% male] and 323 227 from Victoria [49.4% female, 50.5% male]) with NAPLAN test scores in grade 3 (age 8-9 years) and 292 027 adolescents from NSW with test scores in grade 9 (49.3% female, 50.7% male). There were an additional 15 699 and 6688 participants exempt from grade 3 testing in NSW and Victoria, respectively. In the grade 3 cohort, 6.4% of the children in NSW and 7.0% in Victoria were born preterm (23-36 weeks’ gestation) and 54.1% in NSW and 41.1% in Victoria were born at 39 to 40 weeks’ gestation; in the grade 9 cohort, 6.3% were born at 23 to 36 and 53.8% at 39 to 40 weeks’ gestation (Table 1). In NSW, compared with participants born at 39 to 40 weeks’ gestation (n = 418 082 [54.1%]), a higher proportion of participants born at 23 to 27 weeks’ gestation (n = 1319 [0.2%]) had mothers who were aged younger than 25 years at the time of conception (281 [21.3%] vs 71 302 [17.1%]), who had an educational level less than grade 12 (280 [21.2%] vs 66 582 [15.9%]), or who had preeclampsia (139 [10.5%] vs 15 731 [3.8%]), and a higher percentage were their mother’s first birth (662 [5.02%] vs 167 972 [40.2%]) (Table 1 and eTable 1 in Supplement 1). NSW participants born preterm (<37 weeks’ gestation) were more likely than those born at 39 to 40 weeks to be born with congenital anomalies and to experience neonatal morbidity. Similar results were found for the Victorian cohort (eTable 1 in Supplement 1). Maternal characteristics were similar between the 2 states, although compared with NSW, a higher proportion of women in Victoria were aged over 35 years at conception (26.0% vs 21.9%), were from the least disadvantaged socioeconomic quintile (24.7% vs 20.7%), and had a bachelor’s degree or higher (43.6% vs 38.8%) (eTable 1 in Supplement 1).

**Table 1.** Cohort Characteristics in Children and Adolescents With NAPLAN School Results in Grade 3 for NSW and Victoria and Grade 9 for NSW

Characteristic	Participants, No. (%) (N = 1 387 843)^a^		
	NSW grade 3 (n = 772 589)	Victoria grade 3 (n = 323 227)	NSW grade 9 (n = 292 027)
Gestational age at birth, wk			
23-27	1318 (0.2)	526 (0.2)	499 (0.2)
28-31	4589 (0.6)	1476 (0.5)	1784 (0.6)
32-33	6481 (0.8)	2140 (0.7)	2426 (0.8)
34-36	37 191 (4.8)	13 926 (4.3)	13 639 (4.7)
37	45 408 (5.9)	19 189 (5.9)	15 911 (5.4)
38	129 611 (16.8)	51 064 (15.8)	45 471 (15.6)
39-40	418 082 (54.1)	133 002 (41.1)	157 200 (53.8)
41-42	129 909 (16.8)	49 039 (15.2)	55 097 (18.9)
Unknown preterm (<37 wk)	NA	4086 (1.3)	NA
Missing data	NA	48 779 (15.1)	NA
Year of birth			
2001-2004 in NSW or 2005-2008 in Victoria	282 602 (36.6)	207 472 (64.2)	271 491 (93.0)
2005-2007 in NSW or 2009-2012 in Victoria	230 793 (29.9)	115 755 (35.8)	20 509 (7.0)
2008-2011 in NSW	259 194 (33.5)	NA	27 (<0.1)
Sex			
Female	380 841 (49.3)	159 633 (49.4)	143 826 (49.3)
Male	391 748 (50.7)	163 368 (50.5)	148 201 (50.7)
Missing data	NA	226 (0.1)	NA
Maternal age at conception, y			
<25	133 502 (17.3)	42 122 (13.0)	51 310 (17.6)
25-34	469 545 (60.8)	197 138 (61.0)	182 443 (62.5)
≥35	169 343 (21.9)	83 967 (26.0)	58 171 (19.9)
Missing data	199 (<0.1)	NA	103 (<0.1)
Highest parental educational level			
≤Grade 12 or equivalent	133 013 (17.2)	44 795 (13.9)	45 859 (15.7)
Certificate I to IV (including trade certificate)	214 582 (27.8)	79 826 (24.7)	86 194 (29.5)
Diploma or advanced diploma	119 280 (15.4)	50 643 (15.7)	50 292 (17.2)
≥Bachelor’s degree	299 423 (38.8)	142 209 (44.0)	108 130 (37.0)
Not stated or unknown	6291 (0.8)	5754 (1.8)	1552 (0.5)
SES disadvantage, quintile			
1 (Most disadvantaged)	199 569 (25.8)	46 161 (14.3)	75 715 (25.9)
2-4	410 548 (53.1)	196 046 (60.7)	154 916 (53.0)
5	160 165 (20.7)	80 640 (24.9)	60 783 (20.8)
Missing data	2307 (0.3)	380 (0.1)	613 (0.2)
Area of residence			
Major city	601 556 (77.9)	NA	229 232 (78.5)
Regional or remote	168 842 (21.9)	NA	62 235 (21.3)
Missing data	2191 (0.3)	NA	560 (0.2)
Smoking during pregnancy			
Yes	102 915 (13.3)	101 061 (31.3)	40 619 (13.9)
No	66 9674 (86.7)	12 967 (4.0)	251 408 (86.1)
Missing data	NA	209 199 (64.7)	NA
Preeclampsia			
Yes	36 411 (4.7)	10 323 (3.2)	19 542 (6.7)
No	736 178 (95.3)	312 904 (96.8)	272 485 (93.3)
Preexisting or gestational diabetes			
Yes	40 942 (5.3)	18 947 (5.9)	14 333 (4.9)
No	731 647 (94.7)	303 256 (93.8)	277 694 (95.1)
Plurality of birth			
Singleton	749 584 (97.0)	315 247 (97.5)	282 849 (96.9)
Multiple birth	23 005 (3.0)	7980 (2.5)	9178 (3.1)
Nulliparous			
Yes	320 210 (41.4)	139 504 (43.2)	122 233 (41.9)
No	452 379 (58.6)	183 634 (56.8)	169 794 (58.1)
Birth weight for gestational age			
Third to 97th percentile	724 832 (93.8)	256 604 (79.4)	273 366 (93.6)
SGA (<third percentile)	22 014 (2.8)	2808 (0.9)	9151 (3.1)
LGA (>97th percentile)	25 149 (3.3)	9057 (2.8)	9248 (3.2)
Missing data	594 (0.1)	54 758 (16.9)	262 (0.1)
Any major congenital anomaly			
Yes	31 736 (4.1)	7071 (2.2)	13 264 (4.5)
No	740 853 (95.9)	316 156 (97.8)	278 763 (95.5)
Severe morbidity at birth			
Yes	64 237 (8.3)	44 013 (13.6)	21 571 (7.4)
No	668 779 (86.6)	279 214 (86.4)	235 199 (80.5)
Missing data	39 573 (5.1)	NA	35 257 (12.1)

Abbreviations: LGA, large for gestational age; NA, not applicable; NAPLAN, National Assessment Program–Literacy and Numeracy; NSW, New South Wales; SES, socioeconomic status; SGA, small for gestational age.

^a^ Missing data were not included in the analyses for characteristics where the proportion of participants with missing data was less than 2%. Not all proportions sum to the total.

### Full Cohort Analysis

In the full cohort analysis across both states, the mean (SD) numeracy *z*-scores in grade 3 were lowest for children born at 23 to 27 weeks’ gestation, at −0.61 (1.08) for NSW and −0.54 (1.04) for Victoria, while corresponding mean (SD) reading scores were −0.37 (1.09) and −0.23 (1.12), respectively (eTable 2 in Supplement 1). Mean numeracy and reading *z*-scores moved closer to the null with increasing gestational age at birth, with minimal differences by 37 weeks’ gestation or more (eTable 2 in Supplement 1). In both states, between 1.7% and 8.1% of children had numeracy and reading scores 2 SDs below the mean.

**Figure 1.**
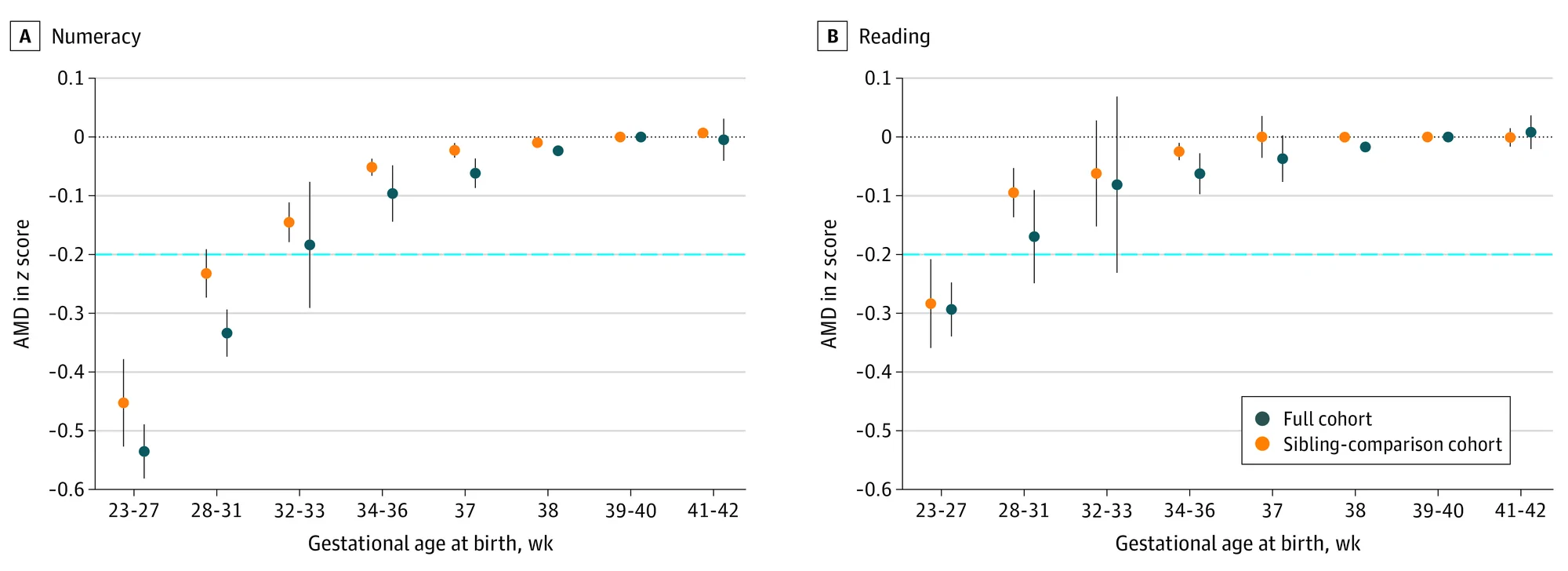
Dot Plots of Pooled Adjusted Mean Differences (AMDs) in Grade 3 Numeracy and Reading *z*-Scores on the National Assessment Program–Literacy and Numeracy Assessment for Children Born at Various Gestational Ages Compared With 39 to 40 Weeks Models adjusted for year of birth, sex, maternal age, smoking during pregnancy, parity, preeclampsia, preexisting or gestational diabetes, highest achieved parental educational level, socioeconomic disadvantage, and remoteness of residence. Dashed blue lines indicate the *z*-score cutoff for a clinically meaningful difference; whiskers, 95% CIs.

**Figure 2.**
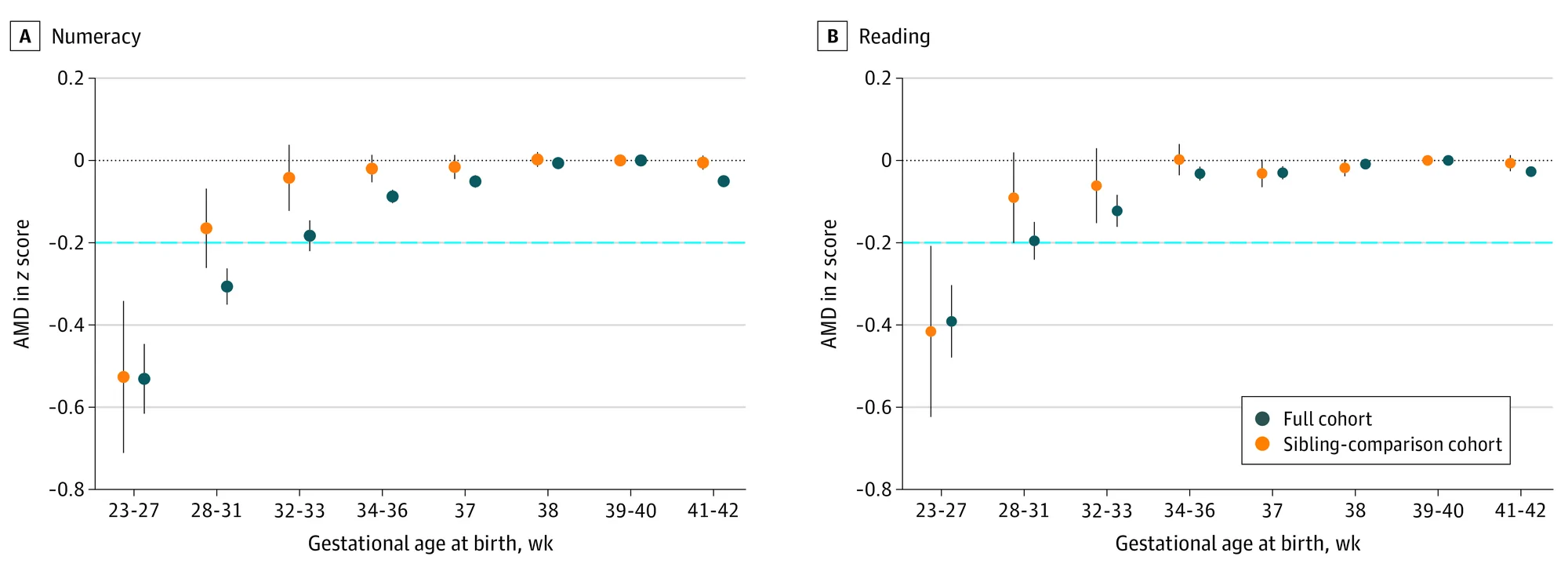
Dot Plots of Adjusted Mean Differences (AMDs) in Grade 9 Numeracy and Reading *z*-Scores on the National Assessment Program–Literacy and Numeracy Assessment for New South Wales Adolescents Born at Various Gestational Ages Compared With 39 to 40 Weeks Models adjusted for year of birth, sex, maternal age, smoking during pregnancy, parity, preeclampsia, preexisting or gestational diabetes, highest achieved parental educational level, socioeconomic disadvantage, remoteness of residence, and *z*-score result in grade 3. Dashed blue lines indicate the *z*-score cutoff for a clinically meaningful difference; whiskers, 95% CIs.

Compared with children born at 39 to 40 weeks’ gestation in the full cohort and after adjusting for covariates, children born at 38 weeks had lower numeracy (AMD, −0.02; 95% CI, −0.03 to −0.02) and reading (AMD, −0.02; 95% CI, −0.02 to −0.01) *z*-scores at grade 3, but the greatest difference in grade 3 numeracy and reading scores was found among children born at 23 to 27 weeks’ gestation for both states (eFigure 1 and eTable 3 in Supplement 1). When the data were pooled, the AMD in *z*-scores for children born at 23 to 27 weeks’ gestation was −0.54 (95% CI, −0.58 to −0.49) for numeracy and −0.29 (95% CI, −0.34 to −0.25) for reading compared with births at 39 to 40 weeks’ gestation (Figure 1). Statistically significant differences compared with birth at 39 to 40 weeks were present for each gestational age group except for 41 to 42 weeks’ gestation (Figure 1 and eTable 3 in Supplement 1). Although differences in scores reduced as gestational age increased, there was no clinically meaningful difference in numeracy scores for children born at or after 34 weeks’ gestation and no clinically meaningful difference in reading scores apparent for those born at or after 32 weeks’ gestation compared with 39 to 40 weeks’ gestation. When assessing numeracy and reading scores in grade 9 among the NSW cohort, we found similar results, with the greatest mean difference in scores found for births at 23 to 27 weeks’ gestation when compared with births at 39 to 40 weeks (numeracy: AMD, −0.53 [95% CI, −0.62 to −0.45]; reading: AMD, −0.39 [95% CI, −0.48 to −0.30]). The adjusted mean difference in scores reduced with increasing gestational age and was not clinically meaningfully different for birth after 32 weeks’ gestation but remained statistically different until birth at 37 weeks’ gestation (Figure 2 and eTable 4 in Supplement 1).

### Sibling Analysis

The sibling analysis included 500 414 individuals in NSW and 155 310 in Victoria, with 6.8% and 7.4% born before 37 weeks’ gestation, respectively (eTable 5 in Supplement 1). When pooling results for both states, birth at less than 37 weeks’ gestation remained associated with lower grade 3 numeracy and reading scores when compared with birth at 39 to 40 weeks (Figure 1 and Table 2). However, this outcome was less pronounced than in analyses performed in the full cohort, and differences compared with children born at 39 to 40 weeks’ gestation were only clinically meaningful for grade 3 numeracy scores among children born at 28 to 31 weeks (AMD, −0.23; 95% CI, −0.27 to −0.19) and 23 to 27 weeks (AMD, −0.45; 95% CI, −0.53 to −0.38), while meaningful differences in reading scores were only observed for children born at 23 to 27 weeks’ gestation (AMD, −0.28; 95% CI, −0.36 to −0.21) (Figure 1 and Table 2). Results for grade 9 numeracy and reading scores revealed that only adolescents born at 23 to 27 weeks’ vs 39 to 40 weeks’ gestation had clinically meaningfully lower scores for numeracy (AMD, −0.53; 95% CI, −0.71 to −0.34) and reading (AMD, −0.42; 95% CI, −0.62 to −0.21).

**Table 2.** Pooled Adjusted Mean Difference in Grade 3 Numeracy and Reading *z*-Scores for Children Born at Various Gestational Ages Compared With 39 to 40 Weeks’ Gestation in Full and Sibling-Comparison Cohorts

Gestational age, wk	Adjusted mean difference (95% CI)^a^			
	Pooled numeracy results		Pooled reading results	
	Full cohort	Sibling cohort	Full cohort	Sibling cohort
23-27	−0.54 (−0.58 to −0.49)	−0.45 (−0.53 to −0.38)	−0.29 (−0.34 to −0.25)	−0.28 (−0.36 to −0.21)
28-31	−0.33 (−0.37 to −0.29)	−0.23 (−0.27 to −0.19)	−0.17 (−0.25 to −0.09)	−0.09 (−0.14 to −0.05)
32-33	−0.18 (−0.29 to −0.08)	−0.15 (−0.18 to −0.11)	−0.08 (−0.23 to 0.07)	−0.06 (−0.15 to 0.03)
34-36	−0.10 (−0.14 to −0.05)	−0.05 (−0.07 to −0.04)	−0.06 (−0.10 to −0.03)	−0.02 (−0.04 to −0.01)
37	−0.06 (−0.09 to −0.04)	−0.02 (−0.04 to −0.01)	−0.04 (−0.08 to 0.00)	0.00 (−0.04 to 0.04)
38	−0.02 (−0.03 to −0.02)	−0.01 (−0.02 to 0.00)	−0.02 (−0.02 to −0.01)	0.00 (−0.01 to 0.01)
39-40	1 [Reference]	1 [Reference]	1 [Reference]	1 [Reference]
41-42	0.00 (−0.04 to 0.03)	0.01 (0.00 to 0.01)	0.01 (−0.02 to 0.04)	0.00 (−0.02 to 0.01)

^a^ Models adjusted for year of birth, sex, maternal age, smoking during pregnancy, parity, preeclampsia, preexisting or gestational diabetes, highest achieved parental educational level, socioeconomic disadvantage, and remoteness of residence; the sibling-adjusted model was further adjusted by the mean gestational age of all siblings in the family.

### Sensitivity Analyses

In supplementary analyses using the full cohort and accounting for outcomes at both the between-family and within-sibling level, results were generally similar to the sibling-comparison model (eFigure 1 in Supplement 1). In the sensitivity analyses when including children exempt from the NAPLAN test, differences were slightly magnified for preterm-birth groups, but the overall trend remained consistent with the results from the main analysis (eFigure 2 in Supplement 1). The association of gestational age with results were consistent when stratifying by sex (eFigure 3 in Supplement 1) and when excluding children born below the third percentile for birth weight or who were multiple births (eFigure 4 in Supplement 1). Similarly, when excluding children with major congenital anomalies, there was no clinically meaningful difference in numeracy results for individuals born after 32 weeks’ gestation vs at 39 to 40 weeks and no clinically meaningful difference in reading results by gestational age (eFigure 5 in Supplement 1). When excluding individuals diagnosed with neonatal morbidity, there was no clinically meaningful difference in NAPLAN results for those born at any gestational age vs at 39 to 40 weeks. In addition, the analysis assessing children below the national minimum standard in numeracy and reading in grade 3 tests showed similar findings (eTable 6 in Supplement 1).

## Discussion

Using 2 statewide Australian birth cohorts linked with educational outcome data for children and adolescents up to 15 years of age, we found that birth at early gestational ages was associated with worse numeracy and reading performance at school. However, our results suggest that these outcome differences, specifically for children born after 32 weeks’ gestation, may be modest and attributed in part to family-level confounding factors. Applying a novel sibling design, we were able to demonstrate that by grade 3 (8-9 years of age), the association between gestational age and educational performance was attenuated, with clinically meaningful differences and lower numeracy scores for children born before 32 weeks’ gestation and lower reading scores for those born between 23 and 27 weeks’ gestation when compared with their siblings born at 39 to 40 weeks’ gestation. By grade 9, there was no clinically meaningful difference in numeracy and reading scores for individuals born after 28 weeks’ gestation compared with 39 to 40 weeks.

The association between gestational age at birth and cognitive and academic performance is well established.^28^ Fetal growth and development are a continuum, with the optimal time of birth at 39 to 40 weeks’ gestation.^29,30,31,32,33^ Preterm birth and disruption in developing organ systems can impair respiratory, metabolic, and neurologic development. Preterm birth also increases the risk of severe neonatal morbidities such as intraventricular hemorrhage, bronchopulmonary dysplasia, and sepsis, which can disrupt normal developmental trajectories.^28,34^ These early biological and environmental perturbations may compromise brain growth and connectivity, predisposing individuals to challenges in cognitive, behavioral, and socioemotional domains as well as reduced educational attainment, mental health, and neurodevelopmental outcomes across the life course.^35,36^

It has been commonly reported by population-based studies that births before 39 to 40 weeks’ gestation are statistically associated with poor educational outcomes for children.^5,6,7,8,9,10,11,12^ Specifically, gestational age has been shown to have an inverse, dose-response pattern whereby children born very preterm (28-32 weeks) and extremely preterm (<28 weeks) have the highest risk, and the risk remains elevated, albeit minimally, for births at up to 38 weeks’ gestation.^5,6,7,8,9,10,11,12^ However, in recent years, with the application of more complex analytical methods, there has been a shift in the evidence, with findings suggesting that these associations may be partly attributable to family-level confounding, factors often unmeasured in traditional cohorts and only assessable when comparing siblings.^15^

As noted, the previous study from Husby et al^15^ used a full sibling analysis to investigate outcomes among individuals aged 15 or 16 years by gestational age at birth and found no difference in cognitive outcomes among those born from 34 to 39 weeks’ gestation. Our study sought to explore earlier educational outcomes to examine whether an association of gestational age at birth with school performance was present earlier in education. Using grade 3 school outcomes (age 8-9 years), we did not observe clinically meaningful differences in numeracy or reading scores for children born from 32 to 38 weeks’ gestation among our sibling analysis. Furthermore, our study looked beyond reporting results based on the strength of the differences between groups, which in previous studies were minimal. Rather, we applied a clinically meaningful cut point to interpret results,^37^ using a threshold value of a difference greater than 0.2 in NAPLAN *z*-scores between gestational age groups; a difference at or above this cut point is considered by Australian education standards as meaningful, with students requiring an educational intervention.^26^

Of note, the sibling analysis was able to control for shared familial characteristics, including unmeasured socioeconomic factors, parental traits, shared genetic factors, and environmental exposures that may confound population-level associations.^38,39^ Recent studies also highlight the complex interplay between genetic predispositions and family environment, suggesting that the expression of educationally relevant genetic potential is moderated by shared genetic risk; parenting quality; mental health; and social, cultural, and religious context.^40,41^ Environmental adversities—including poor housing conditions, exposure to environmental toxins, and limited access to quality schooling—may further compound these effects, perpetuating intergenerational disparities in academic achievement.^38^ Together, these findings demonstrate that family, genetic, and contextual influences may act synergistically to shape educational trajectories across development. Despite the complexity and interaction of these factors, many environmental, social, and health service factors are amenable to interventions that could mitigate them. Given the consistent associations in this study, our findings highlight that early interventions to reduce extremely preterm births and severe neonatal morbidity, combined with long-term follow-up and public health strategies to mitigate adverse outcomes across the continuum of prematurity, may reduce the lifelong impact of preterm birth.

### Strengths and Limitations

Our study was strengthened by the use of 2 large, statewide Australian cohorts totaling over 1 million births. This cohort size allowed us to extend beyond simple classifications of preterm birth (<37 weeks’ gestation) and examine the graded impact of prematurity from children born extremely preterm (23-27 weeks) to those born close to term (35 or 36 weeks). Importantly, the size of our cohort allowed us to deploy a sibling design, with an adequate number of siblings discordant for gestational age at birth to investigate educational outcomes across the spectrum of prematurity while controlling for family effects. This approach allowed us to account for many unmeasured familial and environmental factors that are shared between siblings and that can be very challenging to capture in cohorts using individual, nonsibling births. Analyzing the cohorts separately, we found similar results in both states, which substantially increases the validity of our results. Furthermore, NSW and Victoria are the 2 most populous states in Australia, together accounting for approximately 57% of the country’s population. This means that our results are likely to be broadly generalizable to other Australian states and similar international populations.

There are also some limitations to our study. We assumed that siblings experience shared environmental factors; however, it is plausible that household factors change over the course of birth intervals, and a small proportion of siblings may have been raised in separate households. This is unlikely to have significantly affected our results, given the size of our study cohort. While NAPLAN provides a broad population-level measure of educational achievement, it may not capture subtle or domain-specific learning difficulties that may be detected through detailed neuropsychological assessment. It also does not take into account other factors that may impact performance, such as school attendance, educational practices, and teaching quality, and it may have limited sensitivity for detecting subtle academic differences among children born moderately or late preterm. Additionally, our outcomes used school-based testing, meaning children unable to attend mainstream schooling were not included in our analyses. These exemptions may introduce selection bias; however, our study was not designed to examine outcomes of severe disability or developmental delay but rather educational achievement of mainstream students completing NAPLAN tests. Of note, even with the inclusion of children who were exempt from school testing, the results were similar and consistently highlighted the impact of very early preterm birth. We also cannot exclude residual or unmeasured confounding due to lack of information on other potential covariates such as moving out of state, parental marital status, or children’s body mass index or general health. However, the use of a sibling design allowed us to eliminate many sources of residual unmeasured confounding that may be shared between siblings (including moving out of state, parental marital status, and genetic factors). Additionally, the held Victorian dataset did not include grade 9 outcomes; thus, the findings for the older age group may require further validation.

## Conclusions

In conclusion, in this cohort study pooling results from 2 large cohorts of Australian children and adolescents, our findings highlighted that while birth before 39 weeks’ gestation was associated with lower educational performance in numeracy and reading during early school years, the impact of gestational age was most pronounced among children born before 28 weeks’ gestation. For children born beyond 32 weeks’ gestation, the reduction in numeracy and reading scores was modest and in part may have been attributable to unmeasured shared family factors rather than gestational age at birth. These findings may provide reassurance to parents and clinicians and highlight that further strategies to reduce the rates of preterm birth should particularly focus on those at very preterm (28-31 weeks) and extremely preterm (23-27 weeks) gestational ages and the need for early intervention and support for children born at very or extremely preterm gestation.

## Notes

Supplement 1.
eAppendix
**eTable 1.** Maternal and Neonatal Characteristics by Gestational Age and State in Children Born in New South Wales (NSW) and Victoria **eTable 2.** Mean *z*-Scores and Proportion of Children Significantly Below Normative Numeracy and Reading Scores (<2 SD) by Gestational Age groups in Grade 3 and 9 in Children From New South Wales and Victoria, Australia **eTable 3.** Adjusted Mean Difference in Numeracy and Reading *z*-Scores for Children Born at Various Gestational Ages Compared With 39-40 Weeks’ Gestation for Full, Sibling-Comparison, and Combined Cohort Analyses by State, New South Wales and Victoria **eTable 4.** Adjusted Mean Difference in Grade 9 Numeracy and Reading *z*-Scores for Children Born at Various Gestational Ages Compared With 39-40 Weeks for Full and Sibling-Comparison Cohorts in New South Wales **eTable 5.** Number of Children With and Without a Sibling by Gestational Age and State, New South Wales and Victoria **eTable 6.** Odds of Performing Below the National Minimum Standard in Numeracy and Reading Tests in Grade 3 by Gestational Age Compared With 39-40 Weeks, New South Wales Full and Sibling-Comparison Cohorts **eFigure 1.** Adjusted Mean Differences in Numeracy and Reading Test Scores by Gestational Age for Children in Grade 3 in Full, Sibling-Comparison, and Sibling-Adjusted Cohorts by State, New South Wales and Victoria **eFigure 2.** Adjusted Mean Differences in Numeracy and Reading Test Scores by Gestational Age for Children in Grade 3 in Total Cohort and Including Exempt Children by State, New South Wales and Victoria **eFigure 3.** Adjusted Mean Differences in Numeracy and Reading Test Scores by Gestational Age for Children in Grade 3 Stratified by Sex and State, New South Wales and Victoria **eFigure 4.** Adjusted Mean Differences in Numeracy and Reading Test Scores by Gestational Age for Children in Grade 3 in Total Cohort and Excluding Multiple Births and Births Less Than the Third Percentile for Birth Weight by State, New South Wales and Victoria **eFigure 5.** Adjusted Mean Differences in Numeracy and Reading Test Scores by Gestational Age for Children in Grade 3 in Total Cohort and Excluding Cases of Neonatal Morbidity and Congenital Anomalies by State, New South Wales and Victoria

Supplement 2.
Data Sharing Statement
