# Supplementary material for: Gestational Age and Childhood Educational Outcomes in Primary and High School Years
Source: JAMA Netw Open. 2026 Sep 1;9(9):e2631747. doi: 10.1001/jamanetworkopen.2026.31747 (PMC13535788; doi:10.1001/jamanetworkopen.2026.31747)
Supplement: Supplementary file 1 [file jamanetwopen-e2631747-s001.pdf]

Supplementary Online Content

Schneuer FJ, Lindquist A, Forsythe A, et al. Gestational age and childhood educational outcomes in primary and high school years. *JAMA Netw Open*. 2026;9(9):e2631747. doi:10.1001/jamanetworkopen.2026.31747

eAppendix

eTable 1. Maternal and Neonatal Characteristics by Gestational Age and State in Children Born in New South Wales (NSW) and Victoria

eTable 2. Mean *z*-Scores and Proportion of Children Significantly Below Normative Numeracy and Reading Scores (<2 SD) by Gestational Age groups in Grade 3 and 9 in Children From New South Wales and Victoria, Australia

eTable 3. Adjusted Mean Difference in Numeracy and Reading *z*-Scores for Children Born at Various Gestational Ages Compared With 39-40 Weeks' Gestation for Full, Sibling-Comparison, and Combined Cohort Analyses by State, New South Wales and Victoria

eTable 4. Adjusted Mean Difference in Grade 9 Numeracy and Reading *z*-Scores for Children Born at Various Gestational Ages Compared With 39-40 Weeks for Full and Sibling-Comparison Cohorts in New South Wales

eTable 5. Number of Children With and Without a Sibling by Gestational Age and State, New South Wales and Victoria

eTable 6. Odds of Performing Below the National Minimum Standard in Numeracy and Reading Tests in Grade 3 by Gestational Age Compared With 39-40 Weeks, New South Wales Full and Sibling-Comparison Cohorts

eFigure 1. Adjusted Mean Differences in Numeracy and Reading Test Scores by Gestational Age for Children in Grade 3 in Full, Sibling-Comparison, and Sibling-Adjusted Cohorts by State, New South Wales and Victoria

eFigure 2. Adjusted Mean Differences in Numeracy and Reading Test Scores by Gestational Age for Children in Grade 3 in Total Cohort and Including Exempt Children by State, New South Wales and Victoria

© 2026 Schneuer FJ et al. *JAMA Network Open*.

eFigure 3. Adjusted Mean Differences in Numeracy and Reading Test Scores by Gestational Age for Children in Grade 3 Stratified by Sex and State, New South Wales and Victoria

eFigure 4. Adjusted Mean Differences in Numeracy and Reading Test Scores by Gestational Age for Children in Grade 3 in Total Cohort and Excluding Multiple Births and Births Less Than the Third Percentile for Birth Weight by State, New South Wales and Victoria

eFigure 5. Adjusted Mean Differences in Numeracy and Reading Test Scores by Gestational Age for Children in Grade 3 in Total Cohort and Excluding Cases of Neonatal Morbidity and Congenital Anomalies by State, New South Wales and Victoria

This supplementary material has been provided by the authors to give readers additional information about their work.

© 2026 Schneuer FJ et al. *JAMA Network Open*.

eAppendix

Data source description:

Birth records were obtained from NSW and VIC Perinatal Data Collection (VPDC), state-wide surveillance databases of all births, including information on the mother, pregnancy, birth and baby. Hospital data was obtained from the NSW Admitted Patient Data Collection (APDC), Victorian Admitted Episodes Database (VAED) and Victorian Emergency Minimum Dataset (VEMD). These datasets contain information on all admissions and emergency presentations from public and private hospitals, including diagnosis and procedures coded according to the 10th revision of the International Classification of Diseases, Australian Modification and the Australian Classification of Health Interventions, respectively. Children's school performance was ascertained from the NSW and VIC Department of Education National Assessment Program – Literacy and Numeracy (NAPLAN) conducted in all government, Catholic and Independent schools. Individual records for each child in NSW and VIC were probabilistically linked by the NSW Centre for Health Record Linkage and Centre for Victorian Data Linkage, respectively.

© 2026 Schneuer FJ et al. *JAMA Network Open*.

Table 1. Maternal and neonatal characteristics by gestational age and state in children born in New South Wales (NSW) and Victoria

Maternal and infant characteristics	23-27 weeks n (%)	28-31 weeks n (%)	32-33 weeks n (%)	34-36 weeks n (%)	37 weeks n (%)	38 Weeks n (%)	39-40 weeks n (%)	41-42 weeks n (%)
**NSW (N=772,589)**	**N= 1,318**	**N=4,589**	**N=6,481**	**N=37,191**	**N=45,408**	**N=129,611**	**N=418,082**	**N=129,909**
Year of birth								
2001-2004	473 (35.9)	1736 (37.8)	2348 (36.2)	13307 (35.8)	15575 (34.3)	43947 (33.9)	152047 (36.4)	53169 (40.9)
2005-2007	408 (31.0)	1350 (29.4)	1985 (30.6)	11178 (30.1)	13600 (30)	39503 (30.5)	123615 (29.6)	39154 (30.1)
2008-2011	437 (33.2)	1503 (32.8)	2148 (33.1)	12706 (34.2)	16233 (35.7)	46161 (35.6)	142420 (34.1)	37586 (28.9)
Maternal age								
<25 years	281 (21.3)	868 (18.9)	1148 (17.7)	6447 (17.3)	7518 (16.6)	18230 (14.1)	71302 (17.1)	27708 (21.3)
25-34 years	720 (54.6)	2610 (56.9)	3709 (57.2)	21551 (57.9)	26664 (58.7)	77697 (59.9)	257317 (61.5)	79277 (61)
35 and older	317 (24.1)	1111 (24.2)	1622 (25)	9186 (24.7)	11211 (24.7)	33664 (26)	89339 (21.4)	22893 (17.6)
Highest parental education								
Year 12 or below	280 (21.2)	976 (21.3)	1350 (20.8)	7060 (19)	8044 (17.7)	20569 (15.9)	66582 (15.9)	22279 (17.1)
Certificate I to IV (including trade certificate)	398 (30.2)	1265 (27.6)	1793 (27.7)	10335 (27.8)	12321 (27.1)	33388 (25.8)	109112 (26.1)	36468 (28.1)
Advanced diploma/Diploma	201 (15.3)	657 (14.3)	948 (14.6)	5407 (14.5)	6740 (14.8)	19527 (15.1)	61664 (14.7)	18871 (14.5)
Bachelor degree or above	372 (28.2)	1430 (31.2)	2080 (32.1)	12304 (33.1)	15967 (35.2)	49778 (38.4)	160413 (38.4)	45918 (35.3)
Not stated/Unknown	67 (5.1)	261 (5.7)	310 (4.8)	2085 (5.6)	2336 (5.1)	6349 (4.9)	20311 (4.9)	6373 (4.9)
SES disadvantage								
1 (Most disadvantaged)	364 (27.6)	1258 (27.4)	1770 (27.3)	9779 (26.3)	12111 (26.7)	32334 (24.9)	107555 (25.7)	34398 (26.5)
2,3 and 4	704 (53.4)	2435 (53.1)	3492 (53.9)	19998 (53.8)	24295 (53.5)	68474 (52.8)	220880 (52.8)	70270 (54.1)
5 (Least disadvantaged)	239 (18.1)	876 (19.1)	1201 (18.5)	7301 (19.6)	8871 (19.5)	28467 (22)	88389 (21.1)	24821 (19.1)
Area of residence								
Major Cities	1044 (79.2)	3638 (79.3)	5077 (78.3)	28847 (77.6)	35667 (78.5)	103121 (79.6)	323296 (77.3)	100866 (77.6)
Regional /remote	264 (20.0)	935 (20.4)	1386 (21.4)	8246 (22.2)	9619 (21.2)	26166 (20.2)	93580 (22.4)	28646 (22.1)
Maternal conditions								
Smoking during pregnancy	280 (21.2)	966 (21.1)	1323 (20.4)	6903 (18.6)	7208 (15.9)	17062 (13.2)	52245 (12.5)	16928 (13)

© 2026 Schneuer FJ et al. *JAMA Network Open*.

Gestational hypertension/ Preeclampsia	139 (10.5)	756 (16.5)	985 (15.2)	4373 (11.8)	3901 (8.6)	7419 (5.7)	15731 (3.8)	3107 (2.4)
Pre-existing diabetes	49 (3.7)	295 (6.4)	547 (8.4)	3075 (8.3)	3722 (8.2)	10609 (8.2)	19970 (4.8)	2675 (2.1)
Plurality								
Singleton	1038 (78.8)	3202 (69.8)	4543 (70.1)	29479 (79.3)	39668 (87.4)	124825 (96.3)	416952 (99.7)	129877 (100)
Multiple birth	280 (21.2)	1387 (30.2)	1938 (29.9)	7712 (20.7)	5740 (12.6)	4786 (3.7)	1130 (0.3)	32 (0)
**Infant characteristics**								
Sex								
Male	647 (49.1)	2423 (52.8)	3390 (52.3)	19785 (53.2)	23649 (52.1)	66586 (51.4)	209603 (50.1)	65665 (50.5)
Female	671 (50.9)	2166 (47.2)	3091 (47.7)	17406 (46.8)	21759 (47.9)	63025 (48.6)	208479 (49.9)	64244 (49.5)
Birthweight for gestational age								
3rd-97th centile	1224 (92.9)	4187 (91.2)	5998 (92.5)	34604 (93)	42512 (93.6)	121346 (93.6)	392886 (94)	122075 (94)
SGA <3rd centile	14 (1.1)	114 (2.5)	183 (2.8)	1113 (3)	1324 (2.9)	3767 (2.9)	11573 (2.8)	3926 (3)
LGA >97th centile	78 (5.9)	281 (6.1)	297 (4.6)	1444 (3.9)	1531 (3.4)	4366 (3.4)	13325 (3.2)	3827 (2.9)
Any major congenital anomaly	268 (20.3)	634 (13.8)	560 (8.6)	2111 (5.7)	2164 (4.8)	5400 (4.2)	15625 (3.7)	4974 (3.8)
Severe morbidity at birth	1254 (95.1)	3994 (87)	3906 (60.3)	8703 (23.4)	4450 (9.8)	8412 (6.5)	23992 (5.7)	9526 (7.3)

© 2026 Schneuer FJ et al. *JAMA Network Open*.

Victoria (N=323,227)	23-27 N=526	28-31 N=1476	32-33 N=2140	34-36 N=13926	37 N=19189	38 N=51064	39-40 N=133002	41-42 N=49039	Preterm, unknown GA** N=4086	Missing N=48779
Year of birth										
2005-2008	281 (53.4)	786 (53.3)	1212 (56.6)	8241 (59.2)	10928 (56.9)	27935 (54.7)	75050 (56.4)	30639 (62.5)	4059 (99.3)	48341 (99.1)
2009-2012	245 (46.6)	690 (46.7)	928 (43.4)	5685 (40.8)	8261 (43.1)	23129 (45.3)	57952 (43.6)	18400 (37.5)	27 (0.7)	438 (0.9)
Maternal age category										
<25 years	67 (12.7)	169 (11.4)	264 (12.3)	1597 (11.5)	1813 (9.4)	4455 (8.7)	15981 (12.0)	7128 (14.5)	747 (18.3)	9901 (20.3)
25-34 years	294 (55.9)	861 (58.3)	1248 (58.3)	8168 (58.7)	11269 (58.7)	30495 (59.7)	83579 (62.8)	31528 (64.3)	2241 (54.8)	27455 (56.3)
35+	165 (31.4)	446 (30.2)	628 (29.3)	4161 (29.9)	6107 (31.8)	16114 (31.6)	33442 (25.1)	10383 (21.2)	1098 (26.9)	11423 (23.4)
Highest parental education										
Year 12 or equivalent or below	86 (16.3)	240 (16.3)	303 (14.2)	2056 (14.8)	2625 (13.7)	6656 (13.0)	16458 (12.4)	6039 (12.3)	887 (21.7)	9445 (19.4)
Certificate I to IV	139 (26.4)	385 (26.1)	572 (26.7)	3455 (24.8)	4528 (23.6)	11791 (23.1)	30945 (23.3)	12293 (25.1)	1217 (29.8)	14501 (29.7)
Advanced diploma/Diploma	81 (15.4)	200 (13.6)	368 (17.2)	2275 (16.3)	3062 (16.0)	8089 (15.8)	20816 (15.7)	7761 (15.8)	569 (13.9)	7422 (15.2)
Bachelor degree or above	208 (39.5)	621 (42.1)	858 (40.1)	5901 (42.4)	8644 (45.0)	23635 (46.3)	62538 (47.0)	22107 (45.1)	1341 (32.8)	16356 (33.5)
Not Stated/Unknown	12 (2.3)	30 (2.0)	39 (1.8)	239 (1.7)	330 (1.7)	893 (1.7)	2245 (1.7)	839 (1.7)	72 (1.8)	1055 (2.2)
SES disadvantage										
1 (Most disadvantaged)	85 (16.2)	201 (13.6)	335 (15.7)	1991 (14.3)	2594 (13.5)	6844 (13.4)	18404 (13.8)	7216 (14.7)	693 (17.0)	7798 (16.0)
2, 3, and 4	318 (60.5)	906 (61.4)	1277 (59.7)	8403 (60.3)	11544 (60.2)	30713 (60.1)	80192 (60.3)	29766 (60.7)	2528 (61.9)	30399 (62.3)
5 (Least disadvantaged)	123 (23.4)	*	*	3522 (25.3)	5032 (26.2)	13447 (26.3)	34261 (25.8)	12002 (24.5)	857 (21.0)	10504 (21.5)
Smoking during pregnancy	34 (6.5)	98 (6.6)	136 (6.4)	791 (5.7)	1026 (5.3)	2450 (4.8)	6290 (4.7)	2110 (4.3)	0	32 (0.1)
Missing	288 (54.8)	802 (54.3)	1226 (57.3)	8378 (60.2)	11048 (57.6)	28272 (55.4)	75874 (57.0)	30897 (63.0)	4063 (99.4)	48351 (99.1)
Preeclampsia	62 (11.8)	265 (18.0)	394 (18.4)	1625 (11.7)	1333 (6.9)	1706 (3.3)	2800 (2.1)	624 (1.3)	498 (12.2)	1016 (2.1)

© 2026 Schneuer FJ et al. *JAMA Network Open*.

Infant characteristics										
Sex										
Male	254 (48.3)	779 (52.8)	1162 (54.3)	7512 (53.9)	10114 (52.7)	26241 (51.4)	66457 (50.0)	24120 (49.2)	2111 (51.7)	24727 (50.7)
Female	272 (51.7)	697 (47.2)	978 (45.7)	6414 (46.1)	9075 (47.3)	24823 (48.6)	66545 (50.0)	24919 (50.8)	1975 (48.3)	24052 (49.3)
Birthweight for gestational age										
3rd-97th centile	396 (75.3)	1247 (84.5)	1899 (88.7)	12682 (91.1)	18188 (94.8)	48665 (95.3)	126751 (95.3)	46776 (95.4)	0	0
SGA<3rd centile	*	*	13 (0.6)	136 (1.0)	182 (0.9)	527 (1.0)	1329 (1.0)	607 (1.2)	0	0
LGA>97th centile	*	*	118 (5.5)	888 (6.4)	681 (3.5)	1568 (3.1)	4242 (3.2)	1470 (3.0)	0	0
Missing	104 (19.8)	151 (10.2)	110 (5.1)	220 (1.6)	138 (0.7)	304 (0.6)	680 (0.5)	186 (0.4)	4086 (100.0)	48779 (100.0)
Any major congenital anomaly	215 (40.9)	240 (16.3)	120 (5.6)	498 (3.6)	506 (2.6)	1032 (2.0)	2547 (1.9)	925 (1.9)	222 (5.4)	766 (1.6)
Severe morbidity at birth	433 (82.3)	1138 (77.1)	1181 (55.2)	3574 (25.7)	2390 (12.5)	5133 (10.1)	14955 (11.2)	6580 (13.4)	1567 (38.4)	7062 (14.5)

Missing categories are not included in the analyses for characteristics where the percent missing was <2%. Not all frequencies will sum to the total

*data suppressed due to low counts, ** While specific gestatinal age was missing, ICD10 code for preterm birth was recorded, including: O60* – Preterm labour and delivery, P07.2 - Extreme immaturity (<28w), P07.3 - Other preterm infant

SGA = small for gestational age, LGA large for gestational age

© 2026 Schneuer FJ et al. *JAMA Network Open*.

eTable 2: Mean z-scores and proportion of children significantly below normative numeracy and reading scores (<2SD) by gestational age groups in Grade 3 and 9 in children from New South Wales and Victoria, Australia

	New South Wales				Victoria			
	Numeracy		Reading		Numeracy		Reading	
	Mean z-score (SD)	Z-score < -2SD n (%)	Mean z-score (SD)	Z-score < -2SD n (%)	Mean z-score (SD)	Z-score < -2SD n (%)	Mean z-score (SD)	Z-score < -2SD n (%)
**Year 3**								
**Gestational age**								
23-27 weeks	-0.61 (1.08)	105 (8.1)	-0.37 (1.09)	73 (5.6)	-0.54 (1.04)	30 (5.2)	-0.23 (1.12)	22 (3.8)
28-31 weeks	-0.39 (1.05)	237 (5.2)	-0.25 (1.09)	211 (4.6)	-0.26 (1.07)	56 (3.6)	-0.08 (1.12)	45 (2.9)
32-33 weeks	-0.27 (1.07)	280 (4.4)	-0.20 (1.11)	296 (4.6)	-0.11 (1.03)	38 (1.7)	0.02 (1.09)	55 (2.5)
34-36 weeks	-0.14 (1.07)	1186 (3.2)	-0.11 (1.09)	1315 (3.6)	-0.03 (1.07)	299 (2.1)	-0.01 (1.09)	391 (2.7)
37 weeks	-0.08 (1.08)	1310 (2.9)	-0.08 (1.08)	1388 (3.1)	0.01 (1.06)	403 (2.0)	0.02 (1.09)	517 (2.6)
38 weeks	0.01 (1.08)	3092 (2.4)	0.00 (1.07)	3338 (2.6)	0.04 (1.07)	999 (1.9)	0.03 (1.08)	1334 (2.6)
39-40 weeks	0.03 (1.08)	9380 (2.3)	0.02 (1.07)	10217 (2.5)	0.06 (1.07)	2326 (1.7)	0.06 (1.08)	3110 (2.3)
41-42 weeks	-0.01 (1.06)	3026 (2.3)	0.00 (1.07)	3222 (2.5)	0.07 (1.07)	870 (1.7)	0.08 (1.09)	1126 (2.3)
Unknown preterm	-		-		-0.23 (1.06)	123 (2.9)	-0.15 (1.11)	159 (3.7)
Missing gest. age	-		-		-0.10 (1.08)	1245 (2.5)	-0.11 (1.09)	1670 (3.3)
**Year 9**								
**Gestational age**								
23-27 weeks	-0.59 (0.92)	Not reported	-0.43 (1.1)	22 (4.9)				
28-31 weeks	-0.32 (1.03)	24 (1.5)	-0.21 (1.15)	81 (4.8)				
32-33 weeks	-0.19 (1.01)	19 (0.8)	-0.12 (1.11)	91 (3.9)				
34-36 weeks	-0.08 (0.99)	69 (0.5)	-0.02 (1.08)	401 (3.0)				
37 weeks	-0.03 (1.02)	101 (0.7)	-0.02 (1.06)	451 (2.9)				
38 weeks	0.05 (1.03)	234 (0.5)	0.04 (1.07)	1186 (2.7)				
39-40 weeks	0.05 (1.02)	752 (0.5)	0.05 (1.05)	3677 (2.4)				
41-42 weeks	-0.01 (1.00)	289 (0.5)	0.01 (1.05)	1389 (2.6)				

SD: Standard deviation

© 2026 Schneuer FJ et al. *JAMA Network Open*.

eTable 3: Adjusted mean difference in numeracy and reading z-scores for children born at various gestational ages compared to 39-40 weeks gestation for full, sibling-comparison and combined cohort analyses, by state, New South Wales and Victoria

	Numeracy			Reading		
**Gestational age**	**Full cohort**	**Sibling cohort analysis**	**Combined full cohort sibling analysis**	**Full cohort**	**Sibling cohort analysis**	**Combined full cohort sibling analysis**
**New South Wales**	**Adjusted mean difference (95% CI)**	**Adjusted mean difference (95% CI)**	**Adjusted mean difference (95% CI)**	**Adjusted mean difference (95% CI)**	**Adjusted mean difference (95% CI)**	**Adjusted mean difference (95% CI)**
23-27 weeks	-0.52 (-0.58, -0.47)	-0.44 (-0.52, -0.36)	-0.42 (-0.48, -0.36)	-0.30 (-0.35, -0.25)	-0.28 (-0.37, -0.20)	-0.22 (-0.28, -0.16)
28-31 weeks	-0.35 (-0.38, -0.32)	-0.23 (-0.28, -0.19)	-0.27 (-0.30, -0.23)	-0.21 (-0.24, -0.18)	-0.10 (-0.14, -0.05)	-0.15 (-0.18, -0.11)
32-33 weeks	-0.24 (-0.26, -0.21)	-0.15 (-0.19, -0.11)	-0.18 (-0.21, -0.15)	-0.16 (-0.18, -0.13)	-0.10 (-0.14, -0.06)	-0.11 (-0.14, -0.08)
34-36 weeks	-0.12 (-0.13, -0.11)	-0.05 (-0.07, -0.04)	-0.08 (-0.10, -0.07)	-0.08 (-0.09, -0.07)	-0.03 (-0.04, -0.01)	-0.05 (-0.06, -0.04)
37 weeks	-0.07 (-0.08, -0.06)	-0.03 (-0.04, -0.01)	-0.06 (-0.07, -0.05)	-0.06 (-0.07, -0.05)	-0.02 (-0.03, 0.00)	-0.05 (-0.06, -0.04)
38 weeks	-0.02 (-0.03, -0.02)	-0.01 (-0.02, 0.00)	-0.02 (-0.03, -0.01)	-0.02 (-0.02, -0.01)	0.00 (-0.01, 0.01)	-0.02 (-0.03, -0.02)
39-40 weeks	Reference	Reference	Reference	Reference	Reference	Reference
41-42 weeks	-0.02 (-0.03, -0.02)	0.01 (0.00, 0.02)	-0.02 (-0.02, -0.01)	-0.01 (-0.01, 0.00)	0.01 (0.00, 0.01)	0.01 (0.01, 0.02)
**Victoria**						
23-27 weeks	-0.57 (-0.66, -0.48)	-0.52 (-0.72, -0.32)	-0.41 (-0.51, -0.31)	-0.28 (-0.38, -0.19)	-0.27 (-0.47, -0.07)	-0.11 (-0.21, -0.01)
28-31 weeks	-0.31 (-0.37, -0.26)	-0.25 (-0.36, -0.13)	-0.20 (-0.26, -0.13)	-0.14 (-0.20, -0.08)	-0.09 (-0.20, 0.03)	-0.01 (-0.08, 0.05)
32-33 weeks	-0.13 (-0.18, -0.09)	-0.15 (-0.24, -0.06)	-0.05 (-0.10, 0.00)	0.00 (-0.05, 0.04)	-0.01 (-0.10, 0.08)	0.09 (0.04, 0.14)
34-36 weeks	-0.07 (-0.09, -0.05)	-0.03 (-0.07, 0.01)	-0.02 (-0.04, 0.00)	-0.05 (-0.07, -0.03)	-0.03 (-0.07, 0.00)	0.01 (-0.01, 0.03)
37 weeks	-0.05 (-0.07, -0.04)	-0.01 (-0.04, 0.02)	-0.02 (-0.04, -0.01)	-0.02 (-0.04, -0.01)	0.01 (-0.02, 0.04)	0.01 (-0.01, 0.03)

© 2026 Schneuer FJ et al. *JAMA Network Open*.

38 weeks	-0.03 (-0.04, -0.02)	-0.02 (-0.03, -0.01)	-0.02 (-0.04, 0.00)	-0.02 (-0.03, 0.01)	-0.01 (-0.03, 0.01)	0.00 (-0.01, 0.01)
39-40 weeks	Reference	Reference	Reference	Reference	Reference	Reference
41-42 weeks	0.01 (0.00, 0.03)	0.00 (-0.02, 0.02)	0.00 (-0.02, 0.01)	0.02 (0.01, 0.03)	-0.02 (-0.04, 0.00)	0.00 (-0.01, 0.02)
Unknown preterm birth	-0.21 (-0.25, -0.18)	-0.04 (-0.11, 0.03)	-0.19 (-0.23, -0.16)	-0.13 (-0.17, -0.10)	0.04 (-0.03, 0.11)	-0.11 (-0.15, -0.08)
Missing gestational age	-0.08 (-0.09, -0.07)	-0.01 (-0.03, 0.01)	-0.07 (-0.09, -0.06)	-0.07 (-0.08, -0.06)	0.01 (-0.01, 0.03)	-0.06 (-0.07, -0.05)

Models adjusted for year of birth, sex, maternal age, smoking during pregnancy, parity, preeclampsia, preexisting or gestational diabetes, highest achieved parental education, parity, socio-economic disadvantage and remoteness of residence; Combined full cohort sibling analysis adjusted model is further adjusted by the mean gestational age of all siblings in the family

© 2026 Schneuer FJ et al. *JAMA Network Open*.

eTable 4: Adjusted mean difference in Grade 9 Numeracy and Reading z-scores for children born at various gestational ages compared with 39-40 weeks for full and sibling-comparison cohorts in New South Wales

	Numeracy		Reading	
Gestational age	Adjusted mean difference (95% CI)<br>Full cohort	Adjusted mean difference (95% CI)<br>Sibling cohort	Adjusted mean difference (95% CI)<br>Full cohort	Adjusted mean difference (95% CI)<br>Sibling cohort
23-27 weeks	-0.53 (-0.62, -0.45)	-0.53 (-0.71, -0.34)	-0.39 (-0.48, -0.30)	-0.42 (-0.62, -0.21)
28-31 weeks	-0.31 (-0.35, -0.26)	-0.17 (-0.26, -0.07)	-0.20 (-0.24, -0.15)	-0.09 (-0.20, 0.02)
32-33 weeks	-0.18 (-0.22, -0.15)	-0.04 (-0.12, 0.04)	-0.12 (-0.16, -0.08)	-0.06 (-0.15, 0.03)
34-36 weeks	-0.09 (-0.10, -0.07)	-0.02 (-0.05, 0.01)	-0.03 (-0.05, -0.02)	0.00 (-0.04, 0.04)
37 weeks	-0.05 (-0.07, -0.04)	-0.02 (-0.05, 0.01)	-0.03 (-0.05, -0.01)	-0.03 (-0.07, 0.00)
38 weeks	-0.01 (-0.02, 0.00)	0.00 (-0.02, 0.02)	-0.01 (-0.02, 0.00)	-0.02 (-0.04, 0.00)
39-40 weeks	Reference	Reference	Reference	Reference
41-42 weeks	-0.05 (-0.06, -0.04)	-0.01 (-0.02, 0.01)	-0.03 (-0.04, -0.02)	-0.01 (-0.03, 0.01)

Models adjusted for year of birth, sex, maternal age, smoking during pregnancy, parity, preeclampsia, preexisting or gestational diabetes, highest achieved parental education, parity, socio-economic disadvantage and remoteness of residence

© 2026 Schneuer FJ et al. *JAMA Network Open*.

eTable 5: Number of children with and without a sibling by gestational age and state, New South Wales and Victoria

Gestational age	New South Wales		Victoria	
	Children with a sibling	Without sibling (N= 272,175)	Children with a sibling (N= 155,340)	Without sibling (N= 167,887)
	n (%)	n (%)	n (%)	n (%)
23-27 weeks	791 (0.2)	527 (0.2)	211 (0.1)	315 (0.2)
28-31 weeks	3173 (0.6)	1416 (0.5)	746 (0.5)	730 (0.4)
32-33 weeks	4577 (0.9)	1904 (0.7)	1150 (0.7)	990 (0.6)
34-36 weeks	25,407 (5.1)	11,784 (4.3)	7081 (4.6)	6845 (4.1)
37 weeks	30238 (6.0)	15,170 (5.6)	9459 (6.1)	9730 (5.8)
38 weeks	84,945 (17.0)	44,666 (16.4)	24,629 (15.9)	26,435 (15.8)
39-40 weeks	270,209 (54.0)	147,873 (54.3)	63,059 (40.6)	69,943 (41.7)
41-42 weeks	81,074 (16.2)	48,835 (17.9)	22,734 (14.6)	26,305 (15.7)
Unknown preterm			2242 (1.4)	1844 (1.1)
Missing			24,029 (15.5)	24,750 (14.7)

© 2026 Schneuer FJ et al. *JAMA Network Open*.

eTable 6: Odds of performing below the national minimum standard in numeracy and reading tests in grade three by gestational age compared with 39-40 weeks, New South Wales full and sibling-comparison cohorts

Gestational age	Numeracy		Reading	
	Adj Odds ratio (95% CI) Full cohort	Adj Odds ratio (95% CI) Sibling cohort	Adj Odds ratio (95% CI) Full cohort	Adj Odds ratio (95% CI) Sibling cohort
23-27 weeks	3.06 (2.38, 3.95)	2.40 (1.57, 3.65)	2.23 (1.71, 2.91)	1.90 (1.25, 2.87)
28-31 weeks	2.06 (1.78, 2.4)	1.43 (1.11, 1.86)	1.52 (1.3, 1.78)	1.09 (0.84, 1.42)
32-33 weeks	1.65 (1.44, 1.9)	1.29 (1.04, 1.61)	1.58 (1.38, 1.79)	1.2 (0.96, 1.49)
34-36 weeks	1.27 (1.19, 1.36)	1.03 (0.93, 1.15)	1.29 (1.21, 1.37)	1.11 (1, 1.23)
37 weeks	1.25 (1.18, 1.34)	1.08 (0.98, 1.19)	1.17 (1.1, 1.24)	1.03 (0.93, 1.13)
38 weeks	1.06 (1.01, 1.11)	1.01 (0.95, 1.09)	1.04 (1, 1.09)	0.99 (0.93, 1.06)
39-40 weeks	Reference	Reference	Reference	Reference
41-42 weeks	1.03 (0.98, 1.07)	0.96 (0.89, 1.03)	1.02 (0.97, 1.06)	1.00 (0.94, 1.07)

Models adjusted for year of birth, sex, maternal age, smoking during pregnancy, parity, preeclampsia, preexisting or gestational diabetes, highest achieved parental education, parity, socio-economic disadvantage and remoteness of residence

© 2026 Schneuer FJ et al. *JAMA Network Open*.

eFigure 1: Adjusted mean differences in numeracy and reading test scores by gestational age for children in Grade 3 in full, sibling-comparison and sibling-adjusted cohorts by state, New South Wales and Victoria

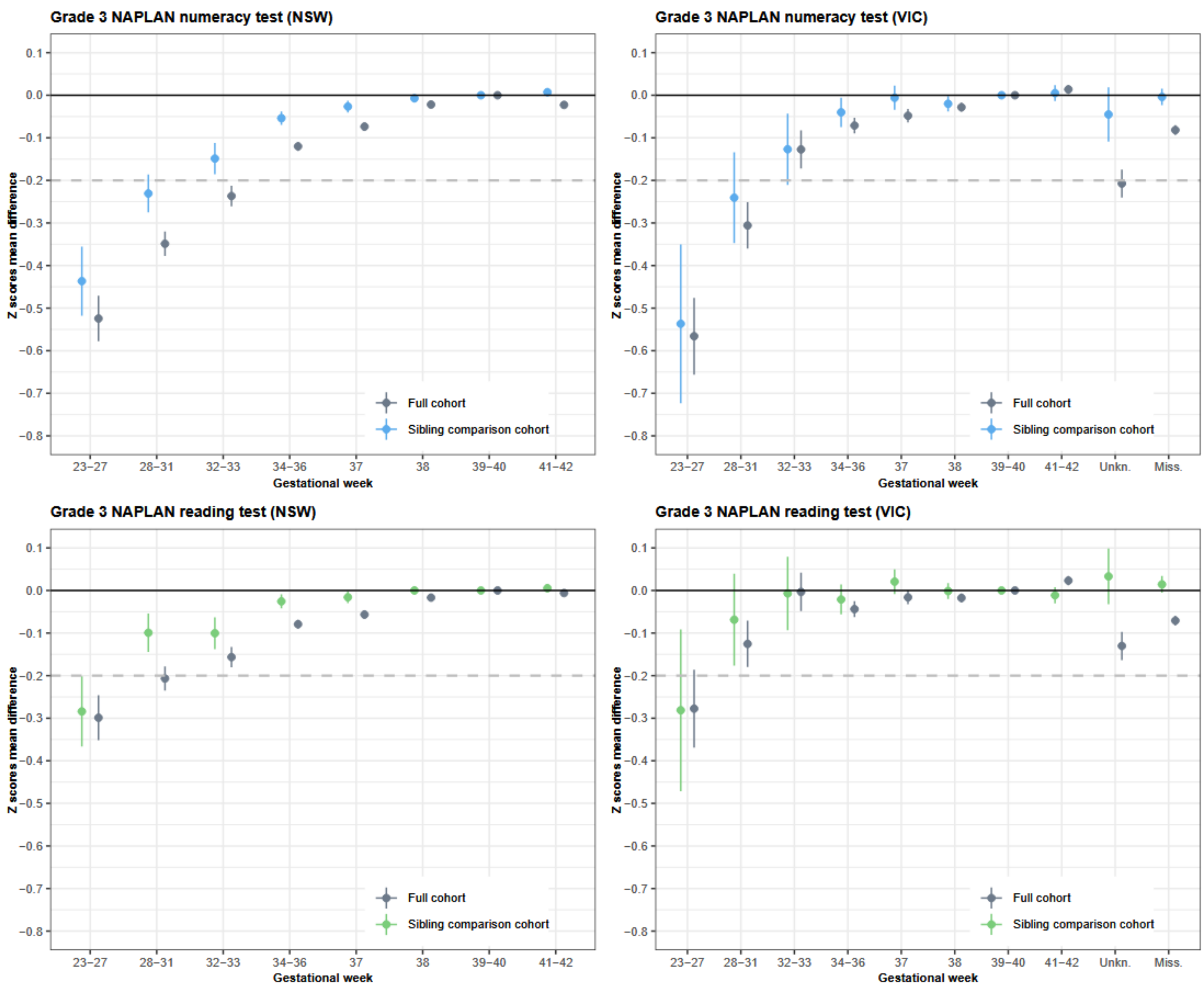

Models adjusted for year of birth, sex, maternal age, smoking during pregnancy, parity, preeclampsia, preexisting or gestational diabetes, highest achieved parental education, parity, socio-economic disadvantage and remoteness of residence

© 2026 Schneuer FJ et al. *JAMA Network Open*.

eFigure 2: Adjusted mean differences in numeracy and reading test scores by gestational age for children in Grade 3 in total cohort and including exempt children by state, New South Wales and Victoria

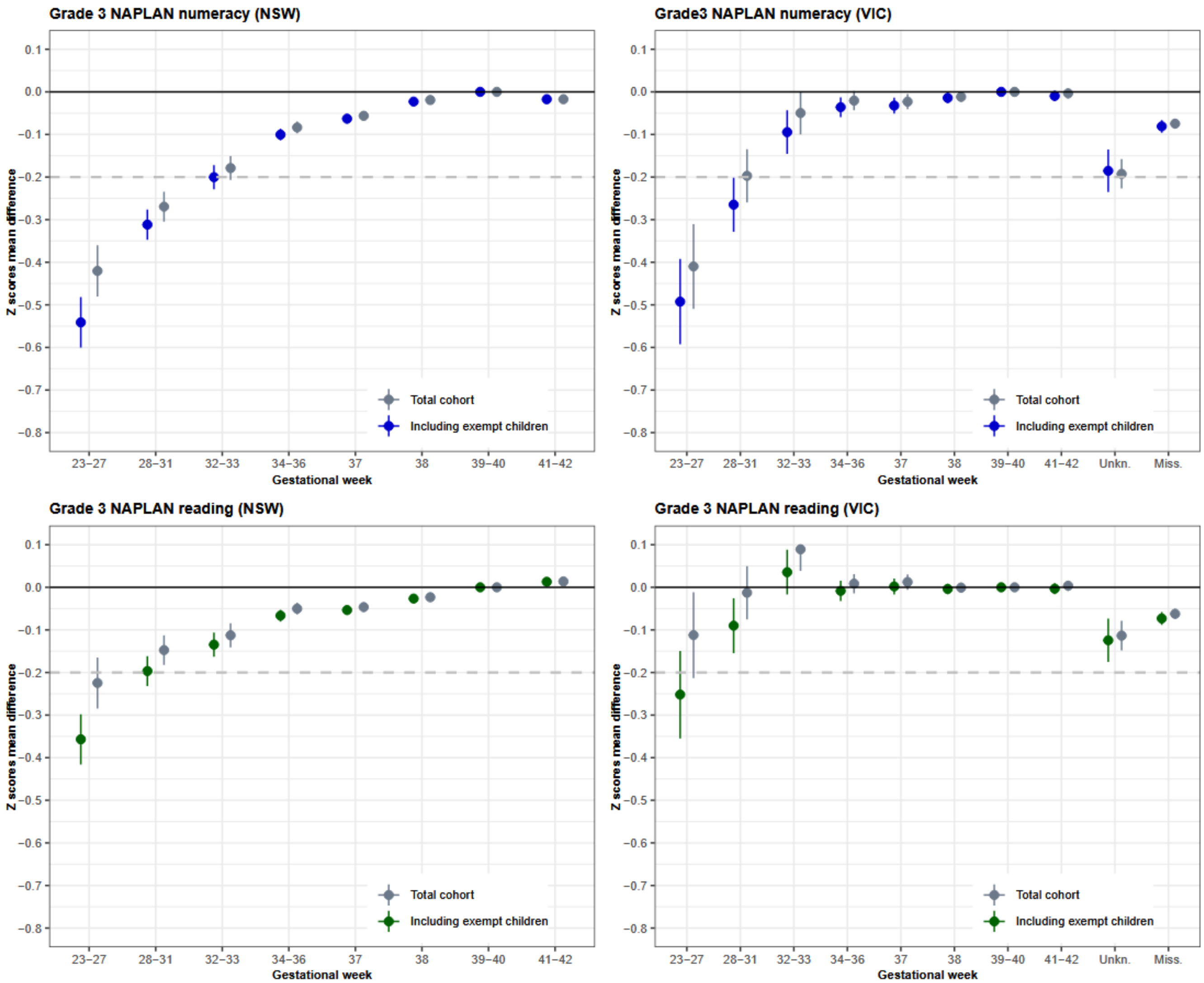

Models adjusted for year of birth, sex, maternal age, smoking during pregnancy, parity, preeclampsia, preexisting or gestational diabetes, highest achieved parental education, parity, socio-economic disadvantage and remoteness of residence

© 2026 Schneuer FJ et al. *JAMA Network Open*.

eFigure 3: Adjusted mean differences in numeracy and reading test scores by gestational age for children in Grade 3 stratified by sex and state, New South Wales and Victoria

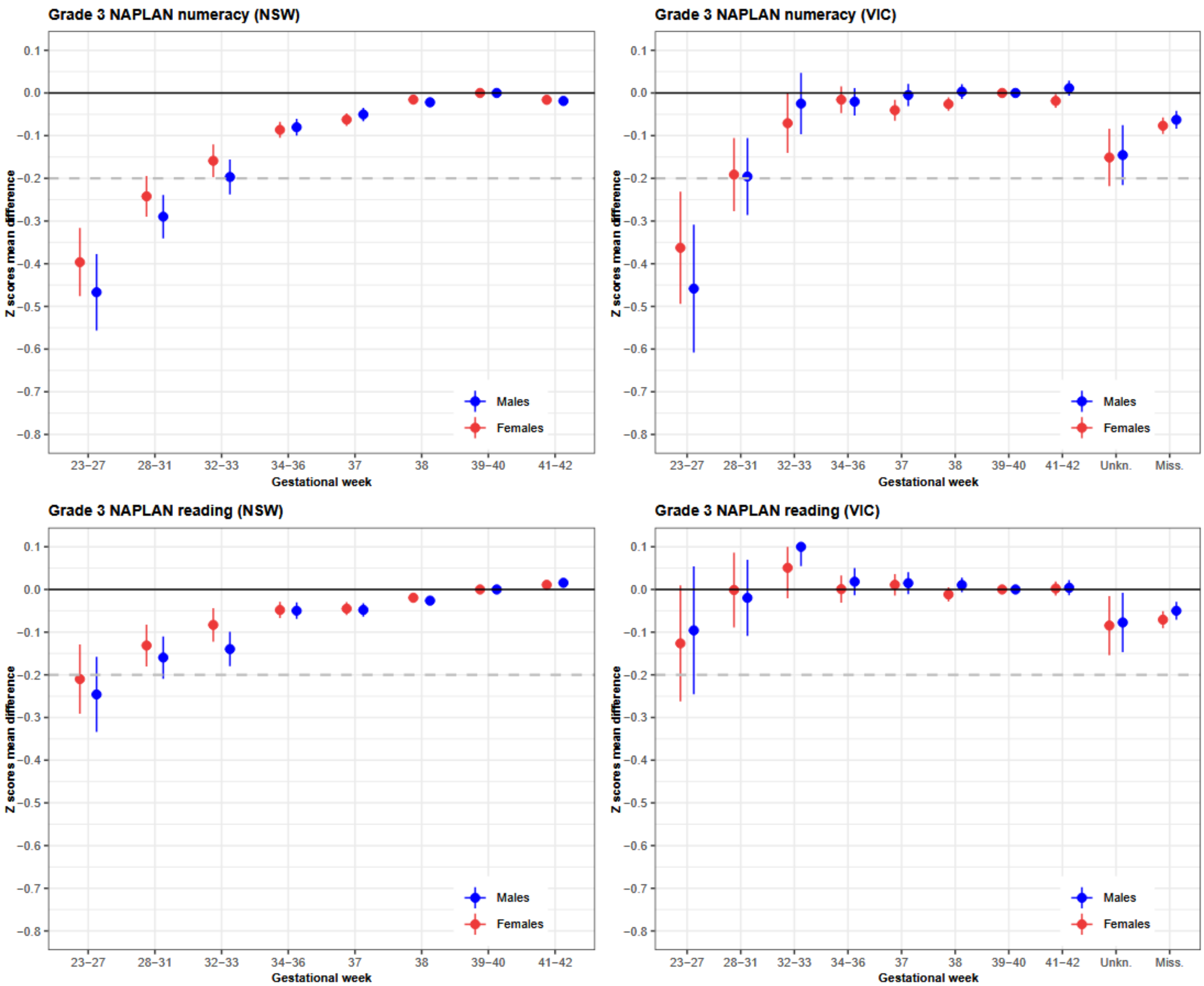

Models adjusted for year of birth, sex, maternal age, smoking during pregnancy, parity, preeclampsia, preexisting or gestational diabetes, highest achieved parental education, parity, socio-economic disadvantage and remoteness of residence

© 2026 Schneuer FJ et al. *JAMA Network Open*.

eFigure 4: Adjusted mean differences in numeracy and reading test scores by gestational age for children in Grade 3 in total cohort and excluding multiple births and births <3rd percentile for birthweight by state, New South Wales and Victoria

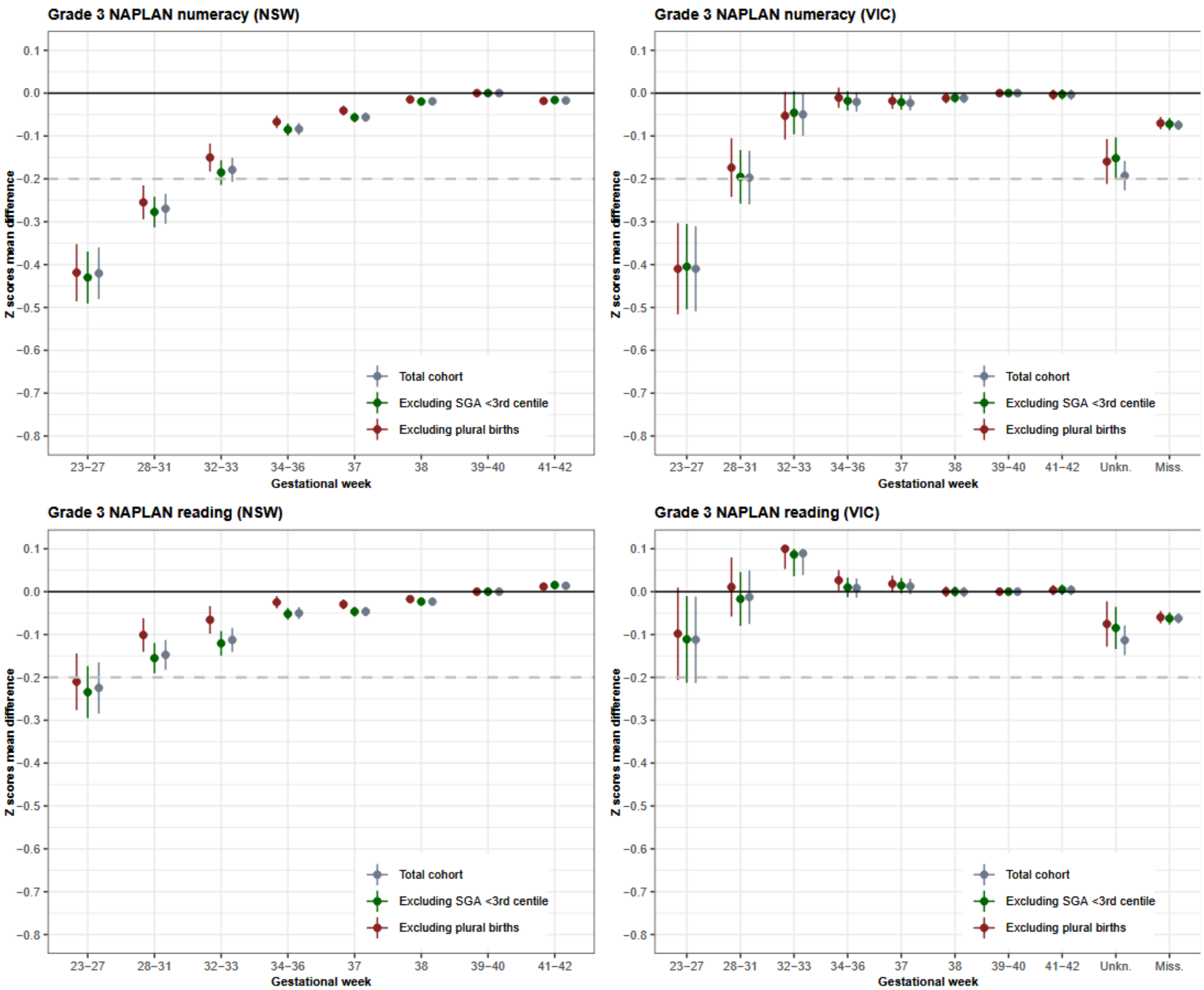

Models adjusted for year of birth, sex, maternal age, smoking during pregnancy, parity, preeclampsia, preexisting or gestational diabetes, highest achieved parental education, parity, socio-economic disadvantage and remoteness of residence

© 2026 Schneuer FJ et al. *JAMA Network Open*.

eFigure 5: Adjusted mean differences in numeracy and reading test scores by gestational age for children in Grade 3 in total cohort and excluding cases of neonatal morbidity and congenital anomalies by state, New South Wales and Victoria

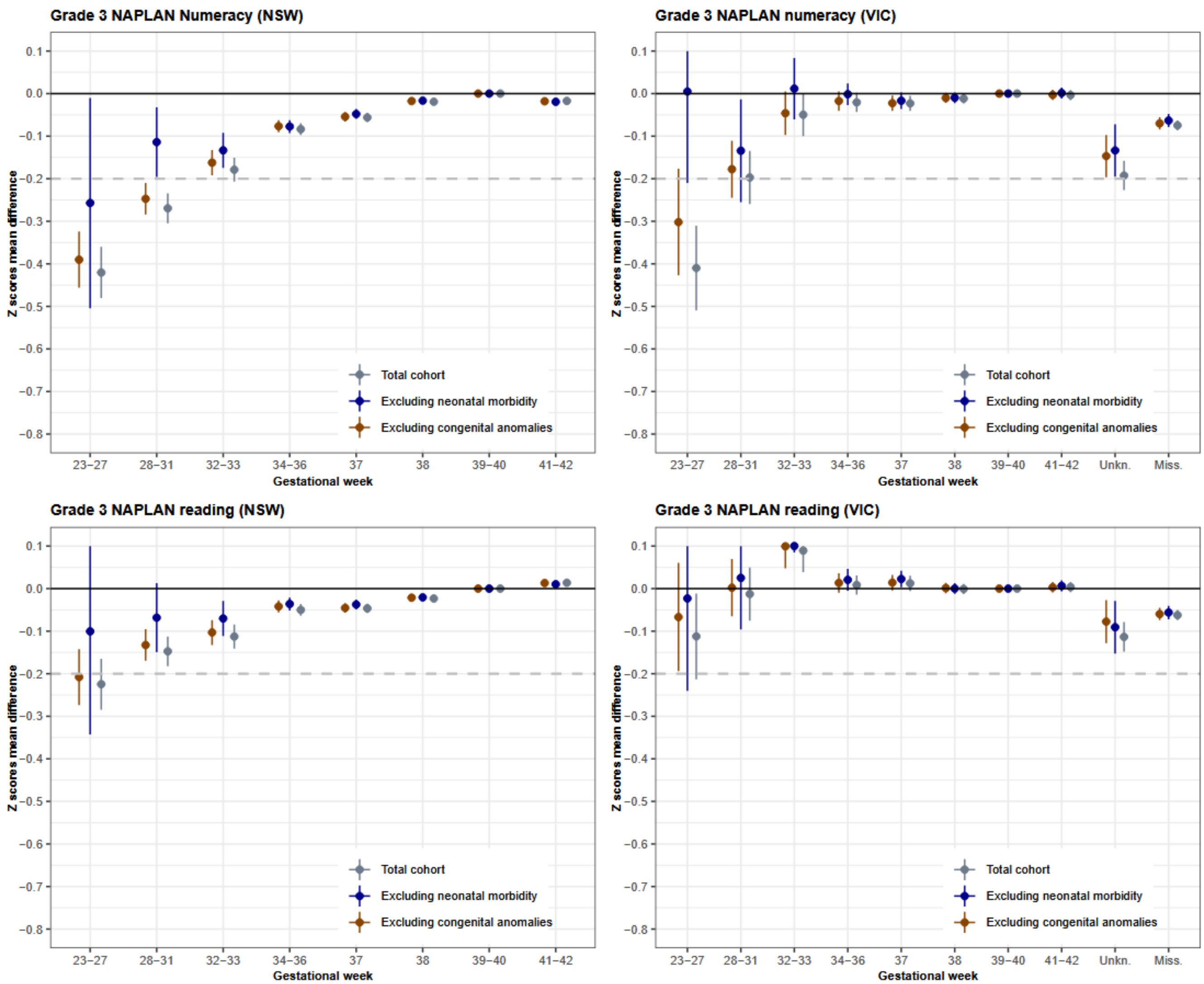

Models adjusted for year of birth, sex, maternal age, smoking during pregnancy, parity, preeclampsia, preexisting or gestational diabetes, highest achieved parental education, parity, socio-economic disadvantage and remoteness of residence

© 2026 Schneuer FJ et al. *JAMA Network Open*.
