# Supplementary material for: Gestational Age and Childhood Educational Outcomes in Primary and High School Years
Source: JAMA Netw Open. 2026 Sep 1;9(9):e2631747. doi: 10.1001/jamanetworkopen.2026.31747 (PMC13535788; doi:10.1001/jamanetworkopen.2026.31747)
Supplement: Supplementary file 2 [file jamanetwopen-e2631747-s002.pdf]

# Data Sharing Statement

Schneuer. Gestational Age and Childhood Educational Outcomes in Primary and High School Years. *JAMA Netw Open*. Published September 01, 2026.
doi:10.1001/jamanetworkopen.2026.31747

**Data**
**Data available:** No

**Additional Information**
**Explanation for why data not available:** Data is available upon request to data custodians
